# Changes in Childhood Immunizations and Intermittent Preventive Malaria Treatment in a Peripheral, Referral Immunization Center During the First 12 Months of COVID-19 Pandemic in Sierra Leone, Western Africa

**DOI:** 10.3389/fped.2022.774281

**Published:** 2022-03-29

**Authors:** Francesco Mariani, Piero Valentini, Matilda Yamba, Abubakar Sidique Turay, Hazel Bulubisi, Umberto Moscato, Francesca Raffaelli, Francesco Iodice, Danilo Buonsenso

**Affiliations:** ^1^Department of Woman and Child Health and Public Health, Fondazione Policlinico Universitario A. Gemelli Istituto di Ricovero e Cura a Carattere Scientifico, Rome, Italy; ^2^Center for Global Health Research and Studies, Università Cattolica del Sacro Cuore, Rome, Italy; ^3^Bureh Town Community Health Center, Bureh Town, Sierra Leone; ^4^Kent Health Post, Kent, Sierra Leone; ^5^Medicine and Surgery, Università Cattolica del Sacro Cuore, Rome, Italy; ^6^Dipartimento Scienze di Laboratorio e Infettivologiche, Fondazione Policlinico Universitario Agostino Gemelli IRCCS, Rome, Italy; ^7^Neurology Unit, Istituto di Ricovero e Cura a Carattere Scientifico S San Raffaele Pisana, Rome, Italy; ^8^Dipartimento di Scienze Biotecnologiche di Base, Cliniche Intensivologiche e Perioperatorie, Università Cattolica del Sacro Cuore, Rome, Italy

**Keywords:** COVID-19, pandemic, vaccines, children immunization, Sierra Leone

## Abstract

**Background:**

There is increasing evidence that the COVID-19 pandemic disrupted childhood immunization services. However, detailed reports on immunizations and preventive antimalarial prophylactic treatments delivered and how the trends changed in referral centers in low-income countries are still missing.

**Methods:**

We performed a retrospective cross-sectional study. Data for vaccinations administered to children <5 years of age, according to the local vaccination schedule, were extracted from the official records of the Kent Community Health Post, Sierra Leone, in the period between April 2019 and March 2021. We compared the vaccinations performed in the first year, considered as a pre-Covid period, with the second year, post-Covid period. Both the period was then divided in four trimester each and the same analysis was operated for each trimester. A Chi-square goodness of fit test was performed to compare the number of vaccinations performed both in the 2 years and in the 8 trimesters.

**Findings:**

Seven thousand two hundred and eighty-three vaccinations were administered: 4,641 in the period between April 2019 and March 2020 and 2,642 between April 2020 and March 2021. The drop in immunizations performed began as soon as the first cases were described in China. The drops were statistically significant when the first three trimesters of the two study periods were compared, while no statistically significant differences were observed for all the vaccines performed in the 4th trimesters. Vaccines administered at birth (BCG) were less affected compared to booster vaccinations.

**Conclusions:**

Immunizations administered in a referral health center in Sierra Leone significantly declined during the pandemic. Although the decline was less pronounced in the last months of the pandemic, we don't think that the small increase would indicate the recovery of previously missed vaccinations. These findings open new public health challenges for the coming years.

## Introduction

Almost 2 years after the first description of a novel Coronavirus pneumonia in China, the world is still facing challenging conditions due to new waves of infections, emergence of new and more contagious variants and inequity in vaccine distribution (1).

In this scenario, children have been relatively spared by the direct effects of the pandemic. Acute SARS-CoV-2 infection in children is rarely severe compared to adults (2), and the Multisystem Inflammatory Syndrome is a rare complication (3) only occasionally fatal (4). Pediatric long covid has been documented but seems less frequent and debilitating (5, 6).

However, the indirect effects of the pandemic on child health have been catastrophic. To limit the spread of the virus, almost all countries worldwide opted for school closures throughout the majority of 2020, disrupting children's routines, scholar, and socialization opportunities, food security and creating a wider disparity in children and family according to baseline socio-economic status of families (7). This in turn led to an increase in mental health issues in children (8). Moreover, hospital reorganizations aimed to offer appropriate care to the surge of COVID-19 cases. People's fears of getting the infection within health services also impaired access to primary prevention services. For example, millions of people worldwide missed cancer screening programs (9) and new diagnoses of latent tuberculosis infections dropped (10). Importantly, a recently published report from the World Health Organization and UNICEF highlighted that, overall, infant immunization coverage dropped to 83% in 2020, leaving 3.7 million more children unvaccinated or under-vaccinated than in 2019^1^. However, this report describes the overall global estimates according to data as reported by countries grouped in main continents and focused on some immunizations as estimates for global trends for the whole vaccines. Similarly, other published studies mainly focused on the early periods of the pandemic and most in high or middle-income countries (11). Conversely, comprehensive reports of all the childhood immunizations and how the trend has evolved for longer periods during the pandemic are still missing. This data is particularly relevant for low resource settings, where immunization programs may represent one of the most important tools to survive during the first year of life (12).

For these reasons, we performed this study aiming to report a monthly trend of all available immunizations in a peripheral, referral childhood immunization center in Kent, a region in the west of Sierra Leone (Western Africa, where infective disease are one of the most important pathologies to face) during the first 12 months of the pandemic, aiming to understand how overall immunization programs were impacted compared with previous years and how administered vaccines changed according to local and global reported SARS-CoV-2 cases.

## Methods

### Setting

This is a retrospective cross-sectional study performed with the aim of understanding how the COVID-19 pandemic impacted on the number of vaccinations performed in a peripheral but referral vaccination center in Sierra Leone.

Data for vaccinations administered to children <5 years of age were extracted from the official records of the Kent Community Health Post (referral from the local community of Kent, Bureh, Checkpoint, Bonga Wharf, and Quarry; estimated population of about 5,000 people) in the period between April 2019 and March 2021, which includes the pre-pandemic period (prPP) (April 2019 to March 2020), and the pandemic period (PP) (April 2020 to March 2021). During this period, the health centers provided the same services offered in the pre-COVID era, attempting not to limit the immunization program.

Data were collected as absolute aggregated numbers by trained local Community Health Officers. These were extrapolated from a hand-made record in which the only absolute number of vaccinations and type of immunizations performed every day are reported, without collecting any personal data of the vaccinated child.

### Vaccinations

Today in Kent, Sierra Leone there are 275 babies (under 4 years of age), 255 little kids (from 5 to 9 years old), 238 children from 10 to 14 years old^2^. In Sierra Leone, the immunization schedule includes^3^ the administration of Bacille Calmette-Guérin (BCG) vaccine and Oral polio vaccine (OPV0) at birth. OPV is then administered at 6, 10, 14 weeks (which we called OPV1, OPV2, and OPV3, respectively). Rotavirus vaccine is administered at 6 and 10 weeks (ROTA1 and ROTA2, respectively), Pneumococcal conjugate vaccine at 6, 10, 14 weeks (PCV1, PCV2, and PCV3, respectively), along with Diphtheria and Tetanus and Pertussis and Haemophilus influenzae and Hepatitis B vaccine (PENTA1, PENTA2, and PENTA3, respectively). Inactivated polio vaccine (IPV) is scheduled at 14 weeks, Yellow fever (YF) at 9 months, Measles and Rubella at 9 and 15 months (which we called MEASLES). Intermittent preventive malaria treatment is administered at 10 and 14 weeks and 9 months of age (IPT1, IPT2, IPT3, respectively).

### Statistical Analyses

Once the written data was collected, they were transferred to an electronic database subsequently used for the analysis using IBM SPSS Statistics 23.0 software (IBM Corporation, Armonk, NY, USA).

A comparison between the number of vaccinations performed in the 2 years was operated. First, we compared the absolute number of vaccinations performed in each year studied and then we divided the study period in 8 trimesters, four trimesters per-year, to compare the number of vaccinations performed in each period. In this way, we matched the same periods of the year between them. A Chi-square goodness of fit test was performed to compare the number of vaccinations performed both in the 2 years and in the 8 trimesters.

To evaluate if there was a difference in the reduction rate of the different vaccines, we compared BCG reduction rate with PENTA1, PENTA2, and PENTA3 reduction rates to analyze if a vaccine performed at birth in Sierra Leone (BCG) suffered a different deflection than the others. The reduction rate was reported as a percentage. A chi square test was performed to evaluate the difference between the BCG and PENTA's reduction rates.

The Bureh Town Community Health Post Review Board approved this study.

## Results

**Figure 1:**
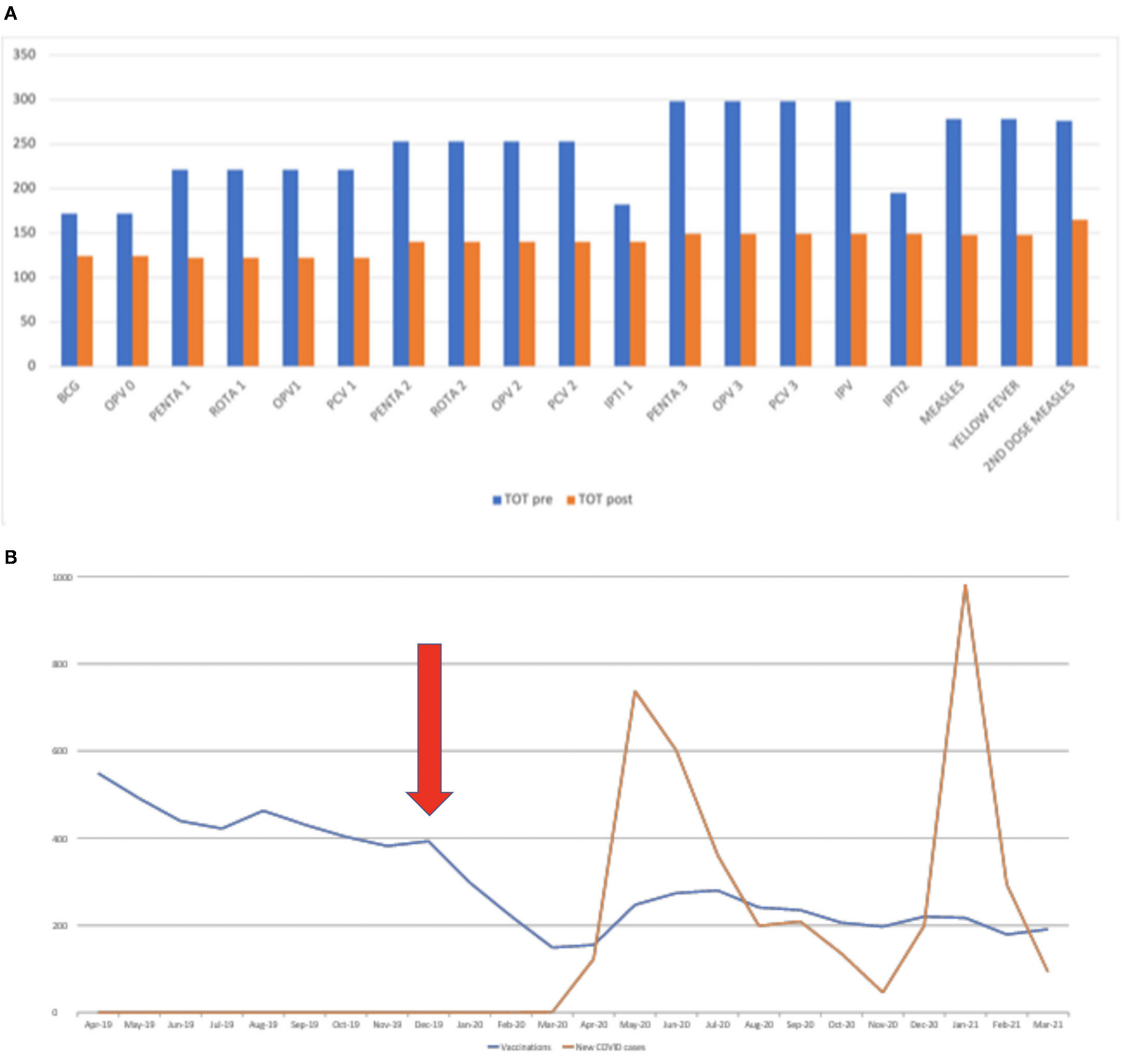
**(A)** Total number of immunizations delivered during the pre- and post-pandemic study periods. The data shows the absolute number of vaccines performed in the first and second year analyzed. In 2020 the number of vaccines performed appears visibly reduced and the different vaccines have undergone different deflections with the less affected which seem to be those performed at birth (BCG and OPV0) and IPTI 1 and 2. **(B)** Overall trend of the administered immunizations according to local SARS-CoV-2 cases and the first detection of SARS-CoV-2 in China. The figure shows how clearly immunizations dropped as soon as the first cases have been notified, before the first cases detected in Sierra Leone. The arrow shows the period in which the first cases were described in China.

During the study period, a total of 7,283 vaccinations were administered: 4,641 in the period between April 2019 and March 2020 and 2,642 between April 2020 and March 2021 (Supplementary Table 1). Overall, the drop observed in the vaccination rate was between 23 and 47% (Figure 1A), with the BCG, OPV0, IPTI1, and IPTI2 sensibly less affected than the others. The number of administered immunizations is shown in Figure 1B, with the arrow pointed on the period in which the first cases in China were described.

**Figure 2:**
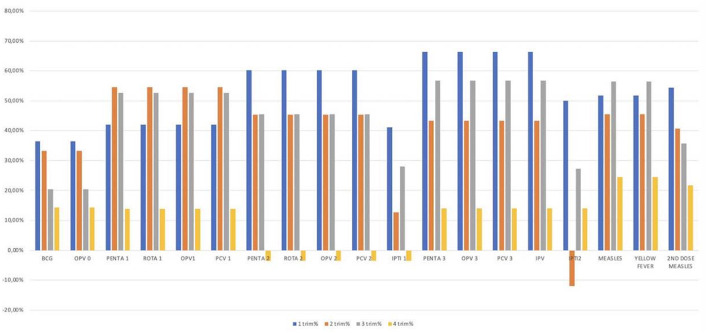
Differences in the number of vaccinations performed per trimester in the 2 years. The figure shows the analysis per trimester; the 2 years studied have been divided in eight trimesters, four per year. The graph shows on the abscissa line the different vaccines studied and on the line of the ordinates the differences in percentage between the vaccines performed in the first and in the second year. Different vaccines showed different deflections with the first and the second trimester most affected for most vaccinations.

To assess how the administered immunization numbers dropped during the study periods, we compared changes according to the same trimester of the two different study periods, as graphically represented in Figure 2 and detailed in Tables 1, 2.

**Table 1:** Differences in the number of vaccinations performed per trimester in the 2 years.

	**Differences 1st trimester**	**Differences 2nd trimester**	**Differences 3rd trimester**	**Differences 4th trimester**
BCG	−36.54%	−33.33%	−20.45%	−14.29%
OPV 0	−36.54%	−33.33%	−20.45%	−14.29%
PENTA 1	−42.03%	−54.55%	−52.63%	−13.79%
ROTA 1	−42.03%	−54.55%	−52.63%	−13.79%
OPV1	−42.03%	−54.55%	−52.63%	−13.79%
PCV 1	−42.03%	−54.55%	−52.63%	−13.79%
PENTA 2	−60.24%	−45.33%	−45.45%	3.45%
ROTA 2	−60.24%	−45.33%	−45.45%	3.45%
OPV 2	−60.24%	−45.33%	−45.45%	3.45%
PCV 2	−60.24%	−45.33%	−45.45%	3.45%
IPTI 1	−41.07%	−12.77%	−28.00%	3.45%
PENTA 3	−66.33%	−43.37%	−56.76%	−13.95%
OPV 3	−66.33%	−43.37%	−56.76%	−13.95%
PCV 3	−66.33%	−43.37%	−56.76%	−13.95%
IPV	−66.33%	−43.37%	−56.76%	−13.95%
IPTI2	−50.00%	11.90%	−27.27%	−13.95%
Measles	−51.72%	−45.45%	−56.52%	−24.44%
Yellow fever	−51.72%	−45.45%	−56.52%	−24.44%
2nd dose measles	−54.43%	−40.74%	−35.71%	−21.74%

*The table shows the percentage of the reduction of the vaccinations performed in the different trimesters*.

**Table 2:** Differences in the number of vaccinations performed per trimester in the 2 years.

	**Differences first trimester**	**Differences second trimester**	**Differences third trimester**	**Differences fourth trimester**	**Total differences**
BCG	19	16	9	4	48
OPV 0	19	16	9	4	48
PENTA 1	29	36	30	4	99
ROTA 1	29	36	30	4	99
OPV1	29	36	30	4	99
PCV 1	29	36	30	4	99
PENTA 2	50	34	30	−1	113
ROTA 2	50	34	30	−1	113
OPV 2	50	34	30	−1	113
PCV 2	50	34	30	−1	113
IPTI 1	23	6	14	−1	42
PENTA 3	65	36	42	6	149
OPV 3	65	36	42	6	149
PCV 3	65	36	42	6	149
IPV	65	36	42	6	149
IPTI2	33	−5	12	6	46
Measles	45	35	39	11	130
Yellow fever	45	35	39	11	130
2nd dose measles	43	33	25	10	111

The analysis of the trimesters highlighted a statistically significant reduction in the vaccination rate in the first trimesters, for all the different vaccines, with a reduction between the 36 and the 66%. The second and the third trimesters showed similar data with a statistically significant deflection in the immunization rate for most of the vaccines (a reduction between the 35 and the 56%). No statistically significant differences were observed for all the vaccines performed in the 4th trimesters (Table 1).

**Figure 3:**
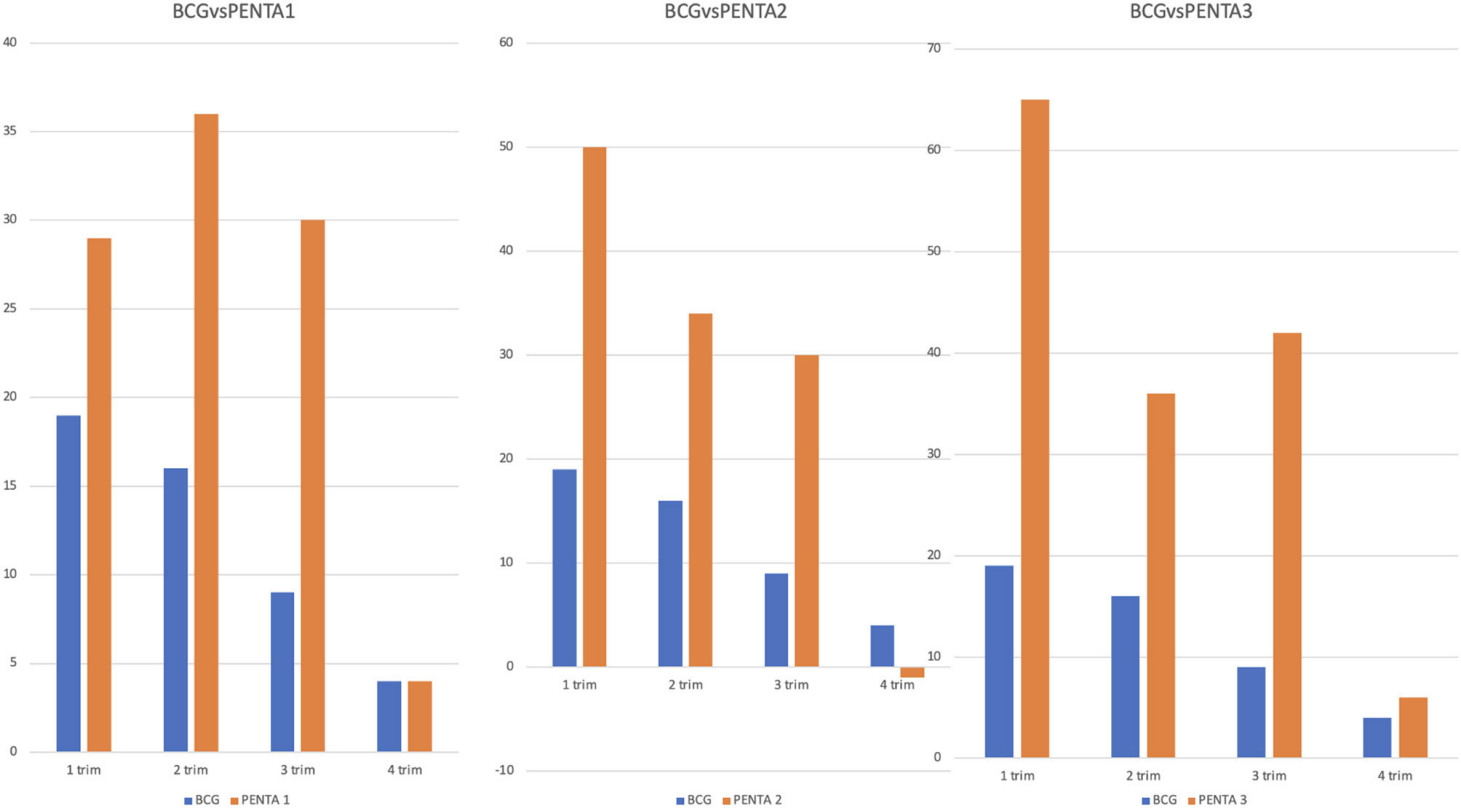
Comparison between BCG and PENTA1, PENTA2, and PENTA3 in the different trimesters. The figure compares the difference between the 2 years of a vaccine performed at birth (BCG) with the difference between the 2 years of three vaccines administered at different time-points (PENTA1, PENTA2, and PENTA3). On the ordinates we reported the differences in absolute number between the vaccines performed in the first and in the second year for each trimester. Although the BCG vaccine shows a generally minor reduction than all the three PENTA, this reduction becomes statistically significant only for the PENTA3 in the first and in the third trimester.

In the hypothesis that vaccines administered at birth may have had a different deflection compared with those administered later during the first 2 years of life, we compared the BCG (administered at birth) with the PENTA vaccines (administered at different time-points). Overall, we found that the BCG vaccine suffered a lower deflection (27%), in the entire study period compared to PENTA1 (44.8%), PENTA2 (44%) and PENTA3 (50%). Although the reduction of the vaccines administered in the two study periods was always higher for all the PENTA vaccines compared to BCG, the differences were particularly relevant and statistically significant when BCG was compared with PENTA3 (p = 0.017, Supplementary Figure 2). We also evaluated how the differences between BCG and the three PENTA vaccines changed according to each trimester of the two study periods (Figure 3; Supplementary Table 2). The reduction of the PENTA vaccines administered compared to BCG was confirmed during the first three trimesters of the two study periods, although the only statistically significative differences were observed between BCG and PENTA3 in the first (*p* = 0.03) and in the third trimester (*p* = 0.04). Conversely, when we analyzed the fourth trimesters of the two periods, reduction rates of BCG and PENTA vaccines were comparable.

Supplementary Figure 1 shows the monthly distribution of all vaccines derived throughout the entire study period while Supplementary Figure 3 shows the comparison between IPT1 and PENTA1.

## Discussion

Although hospital services in Sierra Leone have not undergone any formal limitation during the pandemic, our data showed how the rapid spread of the SARS-COV2 infection, and the fear connected with the possibility of get the infection, have negatively impacted the regular course of vaccination campaigns reducing the uptake of administered vaccinations. In particular, the reduction was particularly marked at the beginning of the pandemic. Unfortunately, even if the trend improved in the last trimester of the study period, there was no full recovery of the missed vaccines or vaccine boosters. Although other studies have described a general decline in vaccination (13) in several countries, to our knowledge this is the first dynamic assessment of how the complete vaccination schedule and intermittent malaria treatments changed during the pandemic in a real-world scenario of an extremely low-resource setting, represented by a referral but peripheral immunization center.

Chiappini et al. (11) recently reported how the population coverage rate for DTP has observed a significant reduction with the advent of COVID infection. Overlapping data were presented by Heneghan et al. in a review published in June 2021 (14), showing a reduction in the rate of vaccinations, more evident in the poorest countries and among the least advantaged social classes. A global joint effort of the World Health Organization and UNICEF also reported how the pandemic severely impacted childhood vaccination (see text footnote ^1^).

Although our preliminary analyses (15) confirmed how this drop in the rate of vaccinations was similarly evident in Sierra Leone, with this work we can see how this reduction has been confirmed over time, assuming a characteristic trend with potential public health implications.

The vaccine drop started evolving over time (Figure 1B). Surprisingly, the reduction in the number of vaccinations performed started well before the first documented cases of COVID-19 in the country. It is therefore interesting to observe that probably it was not the spread of the virus and the subsequent preventive measures established that caused a drop in the immunization rate but rather the fear of people contracting the infection inside health facilities. In fact, we can observe a reduction in the number of vaccinations performed in the same period (December 2019–January 2020) in which the first cases were documented in China.

The subsequent analysis of the trimesters (Figure 2; Table 1) allowed us to observe that, as time passed, the difference in the number of vaccinations performed before and after the beginning of the pandemic has progressively reduced. In particular, compared to an overall statistically significant difference between the two study periods (*p* < 0.0001), in the fourth trimester this difference has lost statistical significance. This trend of progressive reduction of difference is probably attributable to a lower social fear of citizens to go to health facilities and possibly due to better informative campaigns about the importance of not skipping vaccinations.

Although it is true that the data showed an improvement in immunizations administered in the fourth trimester of the second year, we can nevertheless note that there has not been a recovery of missed vaccinations; in fact, an increase in the number of daily vaccines could have been expected following an attempt to recover those not performed in the first year, a fact that was not observed. A recent study estimated a similar trend (13), although this is, to our knowledge, the first documentation of a non-rebound in childhood vaccines administered.

Not all vaccines underwent the same decrease (Figure 2; Table 1). An analysis of the progress of the different vaccines allowed us to note a lower impact of the epidemic on vaccines performed at birth. In this regard, we compared the trend of BCG vaccinations with the PENTA1, PENTA2, and PENTA3 (Figure 3; Supplementary Table 2). Although the statistical analysis showed a statistically significant difference only for the comparison between BCG and PENTA3 in the first and third trimesters of the study periods, it can still be observed that BCG is always less affected than the various PENTAs during each trimester. These data are interesting and can be explained by different hypotheses. First, it shows that the boosters to be made around the year of life have dropped more, suggesting that the population considered the last booster less important and therefore it would have been missed more easily than the other vaccines. The other important fact is that, even if less than the others, vaccines administered at birth have anyway undergone a reduction although the national birth rate of the 2 years remained mostly unchanged^4^. Therefore, it cannot be excluded that, for the same fear of becoming infected in health settings, women preferred giving birth in the villages, unfortunately a more risky but not unusual scenario in such contexts. The normalization of BCG administrations in the last trimester of the study period could indirectly support this hypothesis. This is an important aspect that deserves future investigations and possible preventive interventions, given the great risks associated with home delivery.

Although the evaluation of vaccinations administered was the main purpose of this work, in Sierra Leone the time of vaccination is an opportunity to give children the intermittent malaria prophylactic treatment (IPT), which has undergone a similar reduction, creating the potential indirect consequence of having led to an increase of undiagnosed malaria cases (and subsequent complications) in children in the first year of life. Recent studies have already shown that epidemic (16, 17) and pandemic (18) events have had the indirect consequence of a decline in malaria diagnosis. Considering that many of the malaria deaths are due to diagnostic delays and can occur in more remote villages, this is another important consideration for proactive interventions in the future.

Our study has limitations. First, it is a retrospective study. Second, only aggregated data were collected, and this did not allow us to assess if some factors were associated with greater risk of missed vaccination, such as socioeconomic status, religion or gender. However, due to the emergency period, we have not been able to allocate more resources for a prospective study. Another limitation of the study is that we have no data of the period before March 2019 so it not possible to evaluate if there are differences between the study period and before. Furthermore, in consideration of the fact that we only have aggregated data, it was not possible to build confidence interval. Last, being a referral but peripheral health center, the number of vaccinations administered was relatively small.

In conclusion, the vaccination campaign in Sierra Leone has suffered a significant decline in the last year, mainly in the ^4^ first months of the pandemic. Although the decline was less pronounced in the second half of the pandemic, there was no increase that would indicate the recovery of previously missed vaccinations. The most affected vaccines were those not performed at birth, demonstrating a lower adherence of the population to vaccination campaigns. This aspect opens dangerous scenarios in terms of public health for the following years, given the major impact of vaccinations in the poorest countries, and requires strategies and investments that can fill the hole left by these first months of the pandemic.

## Data Availability Statement

The raw data supporting the conclusions of this article will be made available by the authors, upon request to the corresponding author.

## Ethics Statement

The Bureh Town Community Health Post Review Board approved this study. Written informed consent to participate in this study was provided by the participants' legal guardian/next of kin.

## Author Contributions

All authors listed have made a substantial, direct, and intellectual contribution to the work and approved it for publication.

## Conflict of Interest

MY, FI, and DB were employed by Bureh Town Community Health Center. AT was employed by Kent Health Post. The remaining authors declare that the research was conducted in the absence of any commercial or financial relationships that could be construed as a potential conflict of interest.

## Publisher's Note

All claims expressed in this article are solely those of the authors and do not necessarily represent those of their affiliated organizations, or those of the publisher, the editors and the reviewers. Any product that may be evaluated in this article, or claim that may be made by its manufacturer, is not guaranteed or endorsed by the publisher.

## Footnotes

^1^ https://cdn.who.int/media/docs/default-source/immunization/wuenic—progress-and-challenges-15-july-2021.pdf?sfvrsn=b5eb9141_5&download=true

^2^ https://zhujiworld.com/sl/4008807-kent/

^3^ http://www.vacfa.uct.ac.za/sites/default/files/image_tool/images/210/Immunization_Schedules/Sierra%20Leone.pdf

^4^ https://knoema.com/atlas/Sierra-Leone/Birth-rate
