# Supplementary material for: Changes in Childhood Immunizations and Intermittent Preventive Malaria Treatment in a Peripheral, Referral Immunization Center During the First 12 Months of COVID-19 Pandemic in Sierra Leone, Western Africa
Source: Front Pediatr. 2022 Mar 29;10:774281. doi: 10.3389/fped.2022.774281 (PMC9002134; doi:10.3389/fped.2022.774281)
Supplement: Supplementary file 1 [file Data_Sheet_1.pdf]

SUPPLEMENTARY MATERIAL

	TOT pre<br>#	TOT post<br>#	Difference<br>%
**BCG**	172	124	-27,91%
**OPV 0**	172	124	-27,91%
**PENTA 1**	221	122	-44,80%
**ROTA 1**	221	122	-44,80%
**OPV1**	221	122	-44,80%
**PCV 1**	221	122	-44,80%
**PENTA 2**	253	140	-44,66%
**ROTA 2**	253	140	-44,66%
**OPV 2**	253	140	-44,66%
**PCV 2**	253	140	-44,66%
**IPTI 1**	182	140	-23,08%
**PENTA 3**	298	149	-50,00%
**OPV 3**	298	149	-50,00%
**PCV 3**	298	149	-50,00%
**IPV**	298	149	-50,00%
**IPTI2**	195	149	-23,59%
**MEASLES**	278	148	-46,76%
**YELLOW FEVER**	278	148	-46,76%
**2ND DOSE MEASLES**	276	165	-40,22%

TOT	4641	2642	-43,07%

Supplementary table ST1

The table shows the vaccination performed in the two years of the study. It is possible to notice that all the vaccines have undergone a significant reduction in the second year.

	Difference 1st trimester #	Difference 1st trimester %	Difference 2nd trimester #	Difference 2nd trimester %	Difference 3rd trimester #	Difference 3rd trimester %	Diference 4th trimester #	Diference 4th trimester %
**BCG**	-19	-36,54%	-16	-33,33%	-9	-20,45%	-4	-14,29%
**PENTA 1**	-29	-42,03%	-36	-54,55%	-30	-52,63%	-4	-13,79%
**PENTA 2**	-50	-60,24%	-34	-45,33%	-30	-45,45%	1	3,45%

**PENTA 3**	-65	-66,33%	-36	-43,37%	-42	-56,76%	-6	-13,95%

Supplementary table ST2

The table shows a comparison (in percentage and in absolute number) between the BCG (administered at birth) and the PENTA vaccines (administered at different time-points).

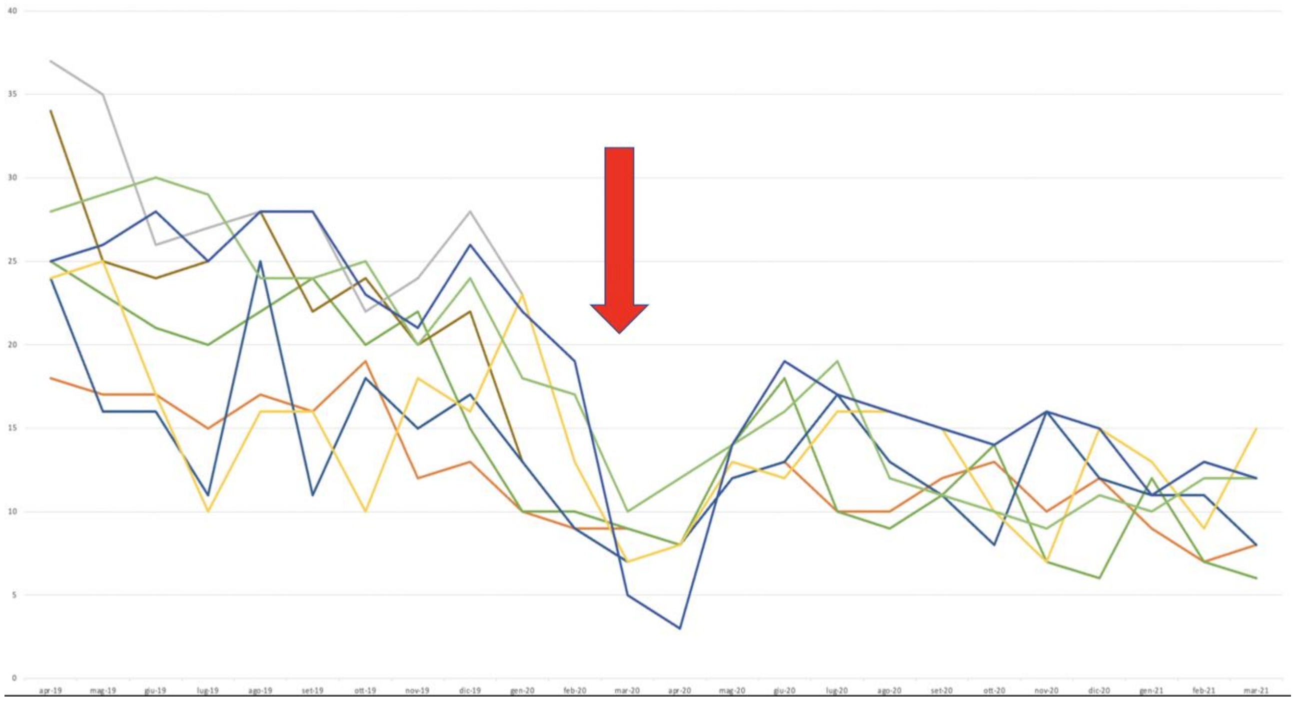

Supplementary figure SF1

The figure shows the monthly distribution of all vaccines derived throughout the entire study period. The arrow shows the passage between pre and post period.

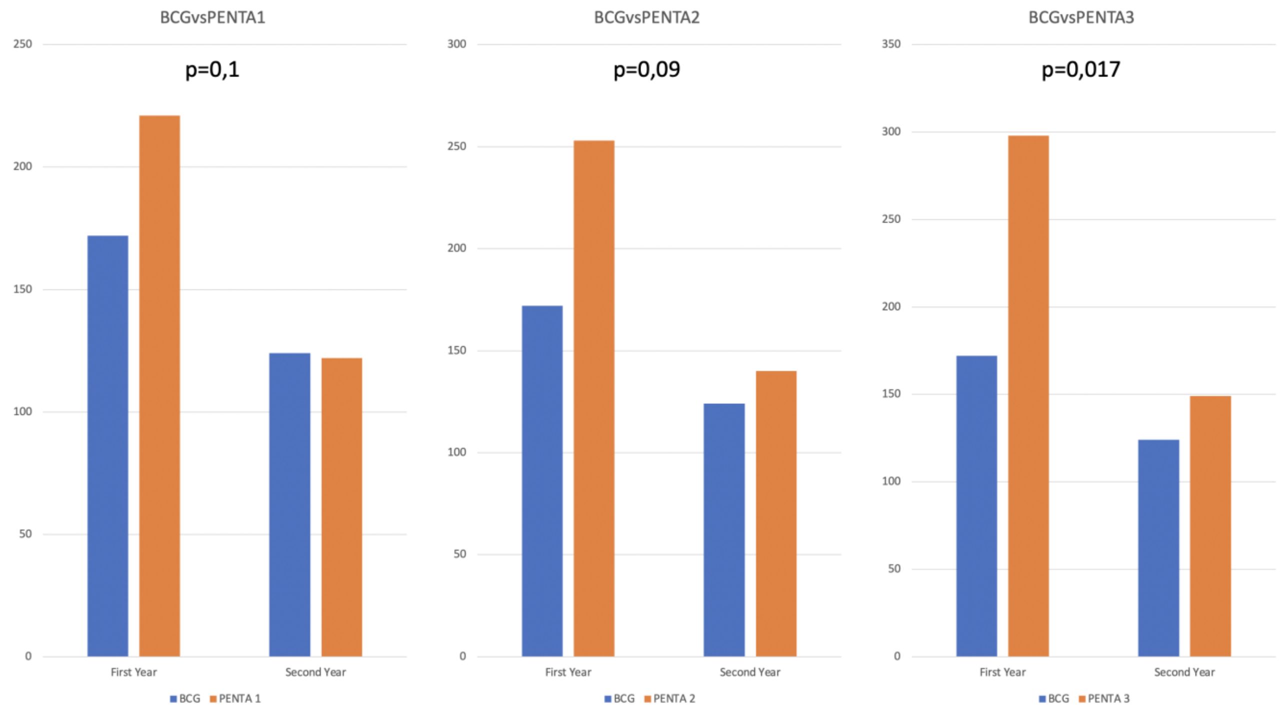

Supplementary figure SF2

The figure shows a comparison between the absolute number of BCG and PENAT1, PENTA2 and PENTA3 vaccinations performed in the two years of the study; the only statistically significant difference may be observed between BCG and PENTA3

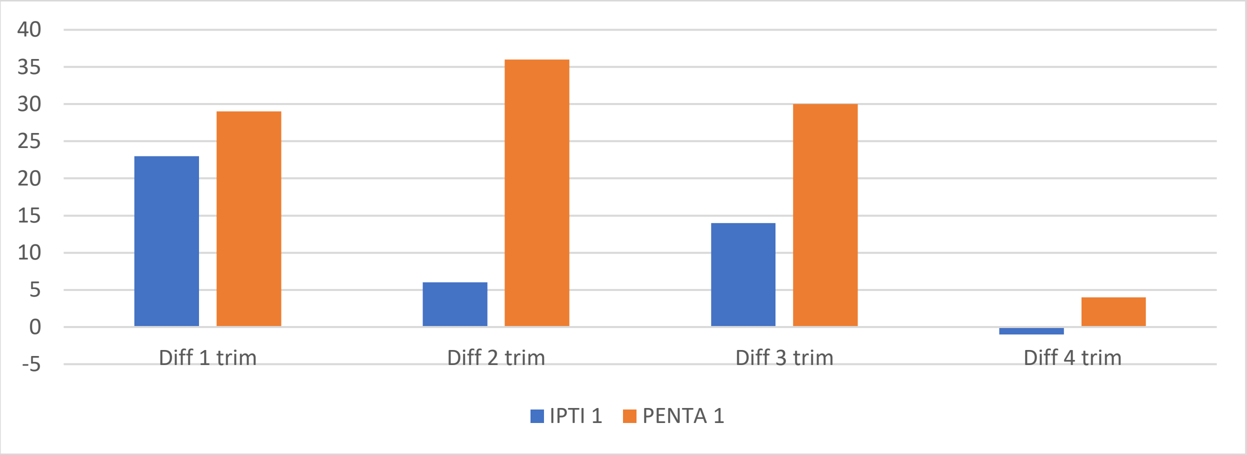

Supplementary figure SF3

The figure shows a comparison between IPT1 and PENTA1 administration in the four trimesters
